# Changes of the liver metabolome following an intravenous lipopolysaccharide injection in Holstein cows supplemented with dietary carnitine

**DOI:** 10.1186/s40104-022-00741-z

**Published:** 2022-08-10

**Authors:** Wei Xu, Sandra Grindler, Ákos Kenéz, Sven Dänicke, Jana Frahm, Korinna Huber

**Affiliations:** 1 Beijing Research Center of Intelligent Equipment for Agriculture, Beijing, 100097 China; 2 Department of Biosystems, Biosystems Technology Cluster, Campus Geel, Kleinhoefstraat 4, 2440 Geel Leuven, KU Belgium; 3 Institute of Animal Science, Faculty of Agricultural Sciences, University of Hohenheim, 70599 Stuttgart, Germany; 4 Department of Infectious Diseases and Public Health, City University of Hong Kong, Block 1, 4/F, To Yuen Building, 31 To Yuen Street, Kowloon, Hong Kong SAR, China; 5 Institute of Animal Nutrition, Federal Research Institute for Animal Health (Friedrich-Loeffler-Institut), 38116 Brunswick, Germany

**Keywords:** Acyl-carnitines, Inflammatory response, Lipid metabolism, Liver metabolome, Mitochondrial function

## Abstract

**Background:**

Carnitine facilitates the flux of long-chain fatty acids for hepatic mitochondrial beta-oxidation, which acts to ameliorate the negative energy balance commonly affecting high-yielding dairy cows. Inflammation triggered by lipopolysaccharide (LPS) load can however pose a challenge to the metabolic integrity via the expression of pro-inflammatory mediators, leading to immune system activation and respective metabolic alterations. The effect of enhanced carnitine availability on hepatic metabolome profiles during an inflammatory challenge has not yet been determined in dairy cows. Herein, Holstein cows were supplemented with 25 g/d rumen-protected carnitine from 42 d prepartum until 126 d postpartum (*n* = 16) or assigned to the control group with no supplementation during the same period (*n* = 14). We biopsied the liver of the cows before (100 d postpartum) and after (112 d postpartum) an intravenous injection of 0.5 µg/kg LPS. Liver samples were subjected to a targeted metabolomics analysis using the AbsoluteIDQ p180 Kit (Biocrates Life Sciences AG, Innsbruck, Austria).

**Results:**

Multivariate statistical analyses revealed that hepatic metabolome profiles changed in relation to both the carnitine supplementation and the LPS challenge. Comparing the metabolite profiles on 100 d, carnitine increased the concentration of short- and long-chain acyl-carnitines, which may be explained by an enhanced mitochondrial fatty acid shuttle and hence greater energy availability. The LPS injection affected hepatic metabolite profiles only in the carnitine supplemented group, particularly altering the concentration of biogenic amines.

**Conclusions:**

Our results point to interactions between an acute hepatic inflammatory response and biogenic amine metabolism, depending on energy availability.

**Supplementary Information:**

The online version contains supplementary material available at 10.1186/s40104-022-00741-z.

## Introduction

Carnitine is a low-molecular-weight compound that was first characterized in muscle extracts. In mammals, carnitine plays important role in lipid metabolism, such as altering the ratio of acetyl-CoA to CoA, shuttling long-chain fatty acids from the cytosol into the mitochondria for subsequent beta-oxidation, and ultimately facilitating energy production [1, 2]. Supplementary carnitine also increased hepatic glucose production in swine by stimulating the flux of gluconeogenic substrates to react with pyruvate carboxylase [2]. In the bovine, supplementary carnitine enhanced the hepatic metabolism of long-chain fatty acids [3]. Metabolic flexibility and adaptation in hepatic lipid and carbohydrate metabolism are desired for optimal health and productivity of dairy cows [4]. Through stimulating the in vitro oxidation of palmitate to acid-soluble products and decreasing the generation of esterified palmitate, supplementary carnitine improved the fat-corrected milk yield [5] and milk fat content [6], which was explained by increased acetyl-CoA availability.

Inflammation during late pregnancy and early lactation is a significant challenge to metabolic health and productive performance in high-yielding dairy cows, linked to central inflammatory responses such as suppressed feed intake and to metabolic alterations triggered by the release of inflammatory mediators such as cytokines and eicosanoids [7–9]. In our previous study, the inflammatory response triggered by lipopolysaccharide (LPS) injection and dietary carnitine supplementation affected plasma and milk metabolite profiles of cows, in particular altering short-chain acyl-carnitine profiles [10]. We speculated that these alterations of circulating acyl-carnitine concentrations originated from changes in hepatic mitochondrial metabolism. Perturbations of plasma acyl-carnitine profiles were proposed to reflect alterations of hepatic function in diabetic human patients [11], and inflammatory signaling through recruitment and activation of Kupffer cells was shown to affect hepatocyte function by local release of cytokines [12]. Besides mitochondrial fatty acid oxidation, the liver is also a central organ for carbohydrate metabolism and for a vast array of further metabolic pathways, such as the synthesis of glutathione via amino acid metabolism, driving antioxidant defense mechanisms [13]. Based on our plasma metabolomics findings, an association between supplementary carnitine and inflammatory response via lipid metabolism was hypothesized [10]. Accordingly, increased carnitine availability affected the expression of genes related to mitochondrial biogenesis and lipid metabolism in the liver of dairy cows challenged by LPS during mid-lactation, as reported in our previous paper [14]. However, no data have been reported yet on the hepatic metabolite profiles of cows treated with supplementary carnitine, particularly during a LPS challenge. Moreover, the effect of supplementary carnitine on productive performance and serum metabolites, such as triglycerides (TG), free fatty acids (FFAs), and β-hydroxybutyrate (BHB), were inconsistent in past studies [15]. Therefore, a comprehensive metabolomics analysis was expected to reveal the hepatic metabolite profiles of cows and the associations between lipid metabolism and inflammatory response in the liver.

Metabolomics aims to detect and quantify the metabolite profiles in biological samples through high-throughput analytical techniques, such as liquid chromatography-mass spectrometry (LC–MS) [16]. Moreover, metabolomics analyses can provide a comprehensive view of mitochondrial metabolism that reflects the functional status of various metabolic pathways [17]. In our previous study, plasma and milk metabolite profiles of the same cows used herein were reported [10]. Metabolomics studies often use surrogate biofluids (such as serum) because the collection of these is relatively less invasive. However, these reflect the sum of appearance and clearance of metabolites in the given biofluid, rather than being organ or process-specific. Therefore, tissue metabotyping is the preferred method when the aim is to better understand underlying mechanisms of organ function and dysfunction [18]. In the current study, we hypothesized that hepatic metabolite profiles could be affected by supplementary carnitine due to enhancing substrate flux for mitochondrial fatty acids oxidation, and that this beneficial effect of carnitine can support oxidative metabolism under the inflammatory response triggered by a LPS injection. Therefore, our objectives were i) to reveal the effect of dietary carnitine supplementation on hepatic metabolite profiles, and ii) to characterize the effect of LPS injection on hepatic metabolite profiles of cows treated with or without supplementary carnitine.

## Materials and methods

### Animals and diet

Animal management and study design, including dietary treatments were reported in our previous study where 54 German Holstein cows were sampled for blood and milk [10]. This study was part of the larger project of the “MitoCow” Consortium, funded by the German Research Foundation. The experiments were conducted at the Institute of Animal Nutrition of the Federal Research Institute for Animal Health (Friedrich Loeffler Institut), Braunschweig, Germany, in accordance with the German Animal Welfare Act and approved by the Lower Saxony Office for Consumer Protection and Food Safety, Oldenburg, Germany (AZ33.19–42,502–04–16/2378). In brief, cows were fed with a partial mixed ration composed of 50% silage (70% maize grass silage, 30% grass silage) and 50% concentrate, according to the recommendation of the Society of Nutrition Physiology (Frankfurt am Main, Germany). Animals were kept in a free stall housing system. All cows were clinically healthy and had free access to water. Cows were randomly assigned to either the control dietary regimen with no additional supplement (CON group) or to the treatment group which was supplemented with 25 g rumen-protected L-carnitine (Kaesler Nutrition GmbH, Cuxhaven, Germany) per day per cow (CAR group). Carnitine was mixed into the concentrate feed. The carnitine treatment commenced on d 44 ± 7 (means ± SD) prepartum and ended on d 126 postpartum. Full details about feeding and housing conditions were published previously [10, 19]. In the current study, the liver samples of 30 cows in mid-lactation were available, including 16 cows in the CAR group and 14 cows in the CON group.

### Lipopolysaccharide challenge

On d 111 postpartum, cows were challenged with standardized LPS by intravenous injection of 0.5 µg *E. coli* LPS per kg body weight (*Escherichia coli* O111:B4, Sigma Aldrich, St. Louis, Missouri, USA). Animals were monitored continuously after LPS administration and clinical observations were published previously [20].

### Sample collection

Liver tissue samples were collected on d 100 (baseline, before LPS injection) and d 112 (24 h after LPS injection) postpartum. Samples of approximately 200 mg were collected by using an automated spring-loaded biopsy instrument (Bard Magnum, Bard, UK) equipped with a 16-gauge needle under local anesthesia (procaine hydrochloride; Isocain 2%, Selectavet, Weyarn-Holzolling, Germany), immediately frozen in liquid nitrogen and stored at −80 °C until further analysis.

Blood serum samples were collected in the morning (at 08:00 h, after the morning milking) from the external jugular vein on d 100 and d 112 postpartum into serum tubes. Blood samples were centrifuged at 2000 × *g* for 15 min at 15 °C to harvest serum. Samples were stored at -80 °C until analysis.

### Measurement of hepatic triglyceride and conventional blood metabolite concentrations

The measurement of hepatic TG was performed by a triglyceride quantification colorimetric kit intended for use in tissue samples (No. K622-100, BioVision, Inc., Milpitas, CA, USA), according to the manufacturer’s protocol, as published previously [14]. The measurement of serum FFA, BHB, and TG were performed by standard colorimetric enzymatic assays using the Eurolyser CCA 180 analyzer (Eurolyser Diagnostica GmbH, Salzburg, Austria), as described in detail previously [20].

### Metabolomics measurement and data pre-processing

The metabolome profiles in hepatic samples were analyzed by using the AbsoluteIDQ p180 Kit (Biocrates Life Science AG, Innsbruck, Austria) as reported previously [10], adapted to tissue extracts. The metabolite extraction from the liver samples was carried out following the standard protocol of the AbsoluteIDQ p180 Kit [21]. Accordingly, liver tissue samples (around 100 mg) were homogenized in ethanol/phosphate buffer (85:15) by mechanical disruption with ceramic beads using a FastPrep-24 5G tissue homogenizer (MP Biomedicals, Inc., Irvine, CA, USA). The supernatants were analyzed by the AbsoluteIDQ p180 Kit, which identifies and quantifies up to 188 metabolites from 5 compound classes: acyl-carnitines (40), proteinogenic and modified amino acids (19), glycerophospho- and sphingolipids (76 phosphatidylcholines, 14 lysophosphatidylcholines, 15 sphingomyelins), biogenic amines (19) and hexoses (1). A detailed list of the compounds is presented in Additional file 1: Table S1. All reagents used in the processing and analysis were of LC–MS grade. The processed concentration data obtained from the LC–MS analysis were first log-transformed, then centered, and Pareto scaled.

### Statistical analyses and data visualization

All statistical analysis and data visualization were performed in an R environment (version 3.5.3). Partial least squares-discriminant analysis (PLS-DA) was performed with the function “plsda()” of the package “MixOmics” to analyze the effects of the LPS challenge on the metabolite profiles. Double cross-validation was performed with the function “mvr_dcv()” in the package “chemometrics”. Important LPS indicators were selected by variable importance in projection (VIP) in the PLS-DA model. Mixed model analysis was performed with the function “lmer()” in the package “lme”, to evaluate the effect of carnitine and LPS and their interactions on the LPS indicators that were identified by high VIP scores, as follows:

$${M}_{jk} = \mu + {LPS}_{j} +{Carnitine}_{k} + {Interaction}_{jk} + {\varepsilon }_{jk}$$

In the equation, *M* represents the hepatic tissue concentration of the metabolites, and *μ* represents the mean. *LPS*_*j*_ represents the fixed class effect of the LPS challenge (*j* = before, after). *Carnitine*_*k*_ represents the fixed class effect of dietary treatment (*k* = control group, carnitine group). *Interaction*_*jk*_ presents the fixed effect of *LPS*_*j*_ × *Carnitine*_*k*_. Cows were considered as a random effect. The model was run again without *Interaction*_*jk*_ if the effect of *Interaction*_*jk*_ was non-significant (*P* > 0.10) in the first run. The random effect can enhance the complexity of the model, resulting in failed convergence of the mixed model. In this case, the linear model was performed with the function “lm()”. The level of significance was determined at *P* < 0.05 in both the mixed model and linear model. The threshold to select important metabolites by the PLS-DA model was VIP > 1.5.

## Results

### Effects of LPS challenge and dietary carnitine on conventional blood metabolites and liver triglycerides

Previously, we reported the concentration of hepatic TG and three serum metabolites (FFA, BHB, and TG) for a larger cohort of cows [14]. Herein, we reported the data subset of the 30 cows involved in the metabolomics analysis and demonstrated the effect of LPS challenge and carnitine on these metabolites, as presented in Table 1. The LPS challenge significantly affected all these 4 variables (*P* < 0.01), while neither of them was affected by the carnitine supplementation. Serum concentrations of FFA and BHB increased after LPS injection, while serum and hepatic concentrations of TG decreased.

**Table 1:** Serum metabolites and hepatic triglyceride concentrations of cows treated with or without dietary carnitine

Metabolites	CAR group		SEM	CON group		SEM	*P*-value		
	Before LPS	After LPS		Before LPS	After LPS		LPS	CAR	Interaction
BHB, μmol/L	0.57	0.79	0.06	0.40	0.88	0.05	< 0.01	0.60	0.05
FFAs, μmol/L	0.14	0.22	0.02	0.12	0.22	0.02	< 0.01	0.91	0.10
Serum TG, μmol/L	0.13	0.11	0.006	0.12	0.12	0.004	< 0.01	1.00	0.06
Hepatic TG, nmol/mg	8.19	5.23	0.62	8.52	5.79	0.70	< 0.01	0.62	NI^1^

^1^*NI* Not included in the mixed model due to the non-significant interaction *P*-value (> 0.10). *CAR* Carnitine-treated, *CON* Control, *BHB* Beta-hydroxybutyrate, *FFAs* Free fatty acids, *TG* triglycerides

‘Before LPS’ refers to lactation d 100, while ‘after LPS’ refers to lactation d 112

### Effects of dietary carnitine on the hepatic metabolite profiles

**Fig. 1:**
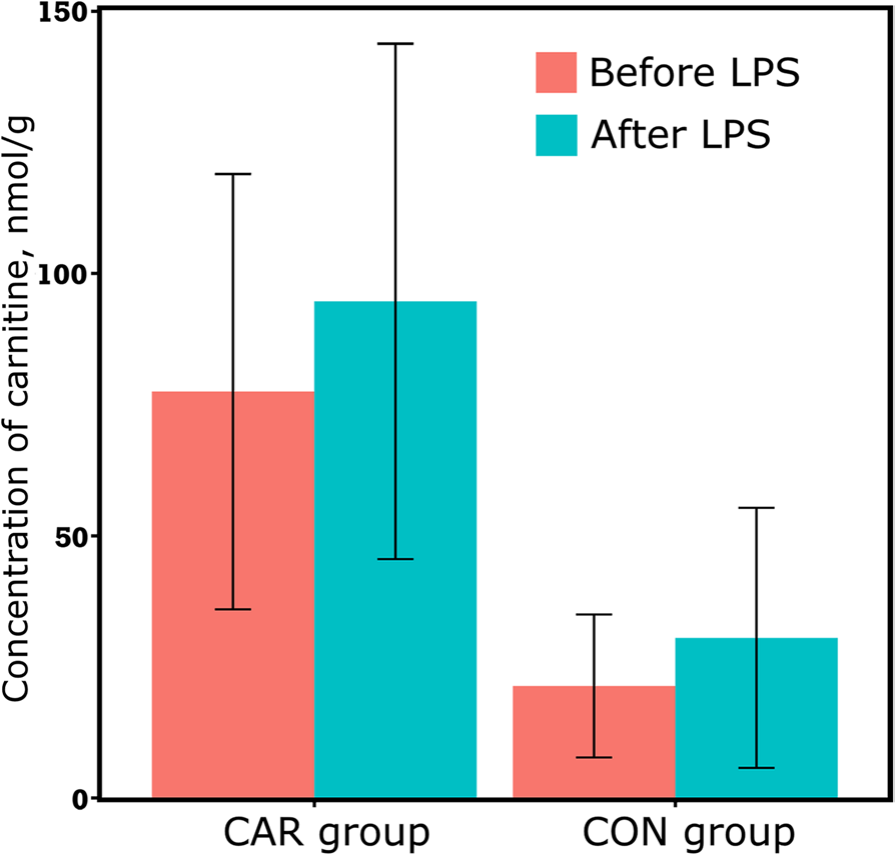
The concentration of hepatic carnitine in dairy cows with (CAR group; *n* = 16) or without (CON group; *n* = 14) supplementary carnitine. Means ± SD, nmol/g (tissue wet weight). LPS effect: *P* = 0.31; Carnitine effect: *P* < 0.01 (mixed model)

**Fig. 2:**
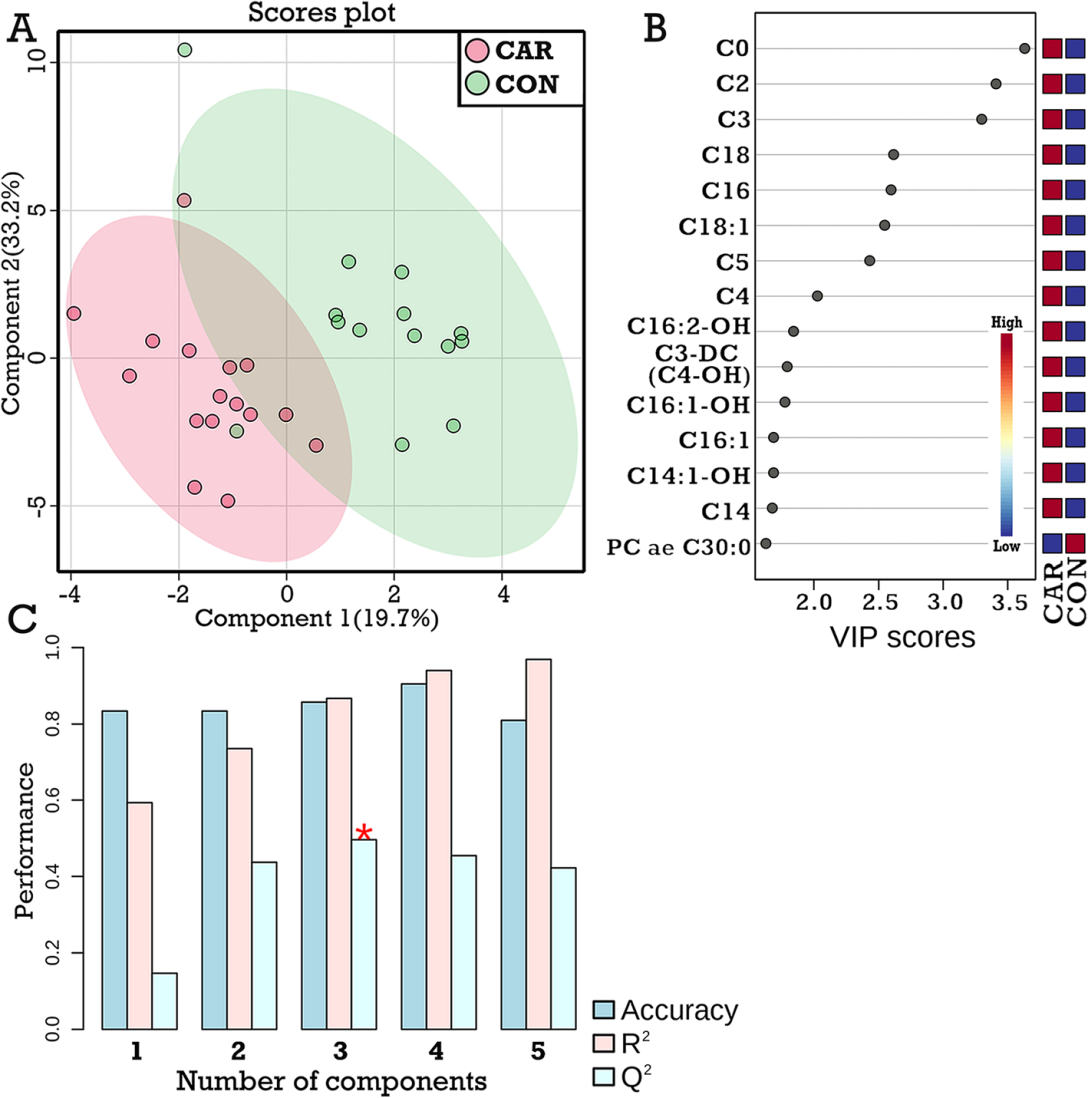
Effect of supplementary carnitine on hepatic metabolome profiles of Holstein cows on d 100 postpartum (before LPS injection). **A,** Hepatic metabolome profiles of cows with (CAR group) or without (CON group) supplementary carnitine by PLS-DA scores plot. **B**, Top 15 important hepatic metabolites of the first PLS-DA component. **C,** The accuracy, the goodness of fit (R^2^), and predictive ability (Q^2^) of the PLS-DA model as assessed by cross-validation. In plot B, the blue and red squares indicate lower and higher metabolite concentrations, respectively. Cx: Acylcarnitines of x chain length; PC: Phosphatidylcholine; SM: Sphingomyelin; lysoPC: Lysophosphatidylcholine. The specific abbreviations of these metabolites are available in Additional file 1: Table S1

Based on our metabolomics assay, the hepatic carnitine concentration was significantly greater in CAR cows than in CON cows (Fig. 1). Based on the hepatic tissue samples collected on d 100, metabolite profiles were different between the CON and the CAR groups, as shown in the PLS-DA scores plot (Fig. 2A). In total, 16 metabolites were classified as important indicators (VIP scores > 1.5) in the first component of the PLS-DA model (Fig. 2B), including carnitine, 4 short-chain acyl-carnitines (C2, C3, C4, and C5), and 3 long-chain acyl-carnitines (C14, C16, and C18). Furthermore, the derivatives of the above acyl-carnitines, such as C4-OH, C16:1-OH, and C18:1, had high VIP scores, too. All these acyl-carnitines and their derivatives had higher concentrations in the CAR group, compared with the CON group. The predictive ability (Q^2^) of the PLS-DA model to discriminate hepatic metabolite profiles of cows with or without supplementary carnitine was 0.57 (Fig. 2C). This was assessed by cross-validation on a scale where a value of Q^2^ = 1.0 indicates perfect discrimination between two groups.

### Effects of LPS challenge on the hepatic metabolite profiles in the CON group

**Fig. 3:**
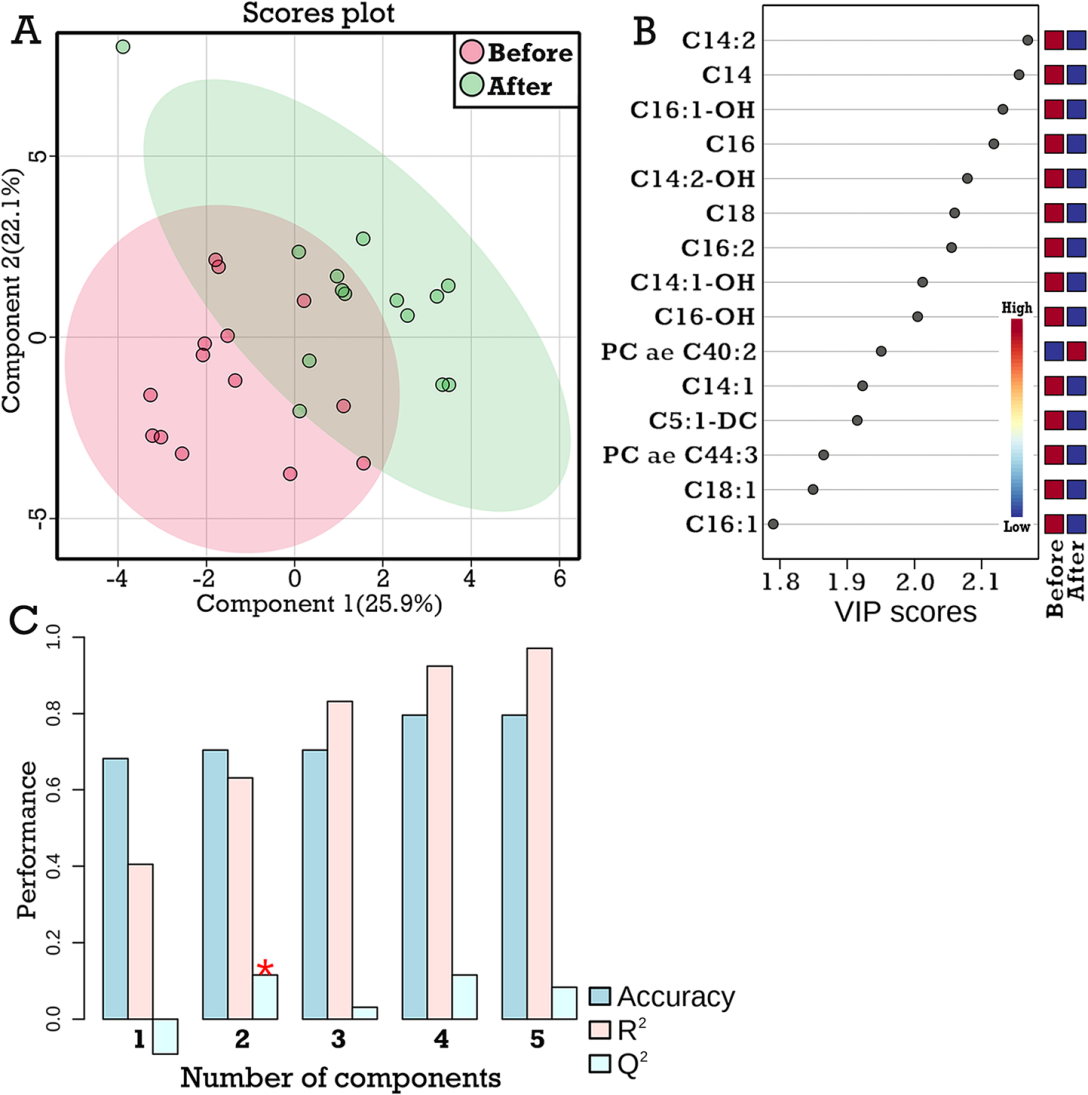
Effect of LPS challenge on the hepatic metabolome profiles of Holstein cows without supplementary carnitine (CON group on d 112 postpartum). **A,** Hepatic metabolome profiles before and after LPS challenge by PLS-DA scores plot. **B**, Top 15 important hepatic metabolites in the first PLS-DA component. **C,** The accuracy, the goodness of fit (R^2^), and predictive ability (Q^2^) of the PLS-DA model as assessed by cross-validation. In plot B, the blue and red squares indicate lower and higher metabolite concentrations, respectively. Cx: Acylcarnitines of x chain length; PC: Phosphatidylcholine; SM: Sphingomyelin; lysoPC: Lysophosphatidylcholine. The specific abbreviations of these metabolites are available in Additional file 1: Table S1

Figure 3A presents the overlapping hepatic metabolite profiles of the CON cows before and after the LPS challenge. Even though no significant separation was observed between the metabolite profiles in the scores plot of PLS-DA, the metabolites with the highest VIP scores were mostly long-chain acyl-carnitines and their derivatives, having decreased concentrations after the LPS injection than before (Fig. 3B). This PLS-DA model had weak metrics as the Q^2^ was not higher than 0.20 (Fig. 3C).

### Effects of LPS challenge on the hepatic metabolite profiles in the CAR group

**Fig. 4:**
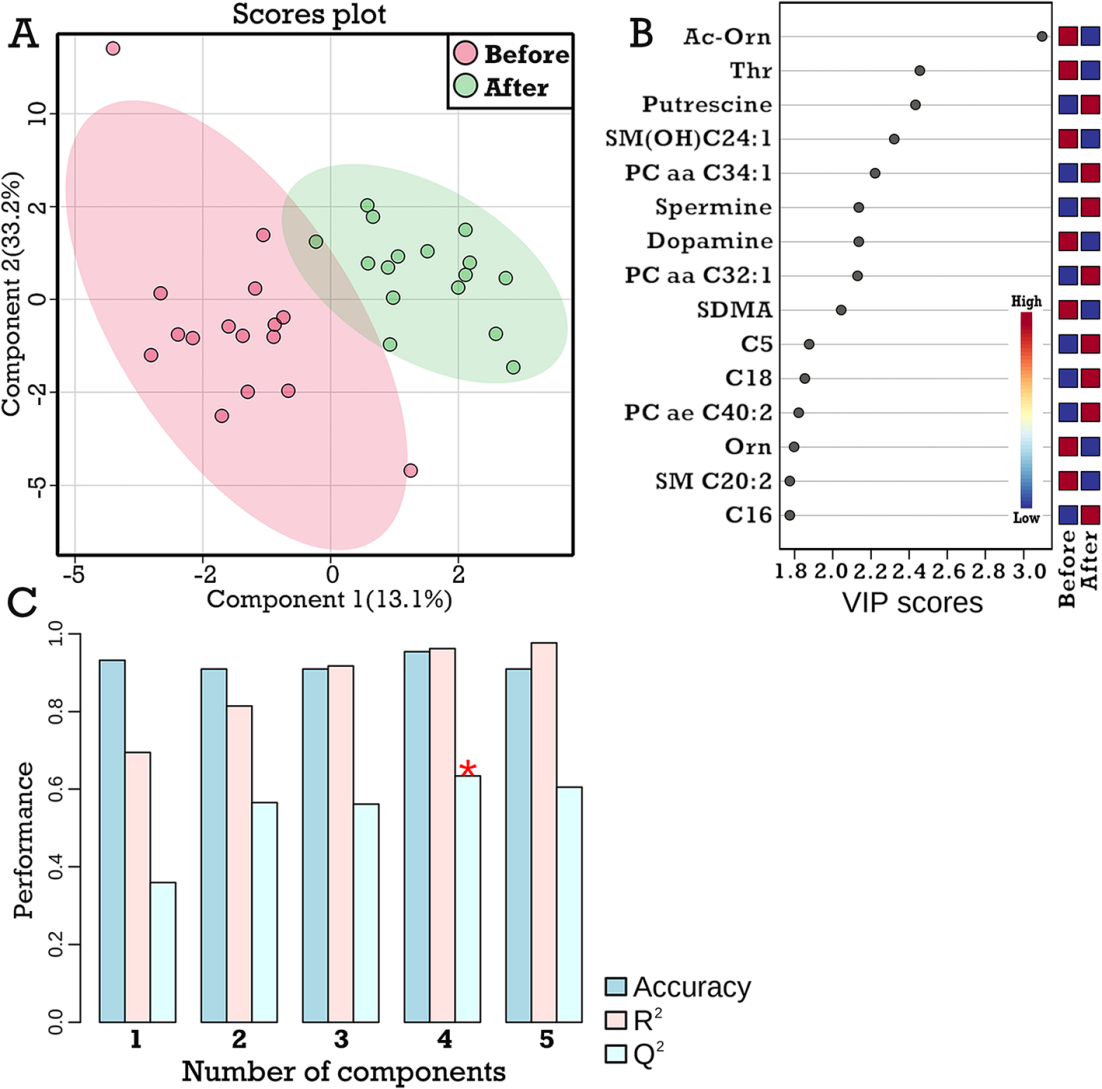
Effect of LPS challenge on the hepatic metabolome profiles of dairy cows with supplementary carnitine (CAR group on d 112 postpartum). **A,** Hepatic metabolome profiles before and after LPS challenge by PLS-DA scores plot. **B**, Top 15 important hepatic metabolites in the first PLS-DA component. **C,** The accuracy, goodness of fit (R^2^), and predictive ability (Q^2^) of the PLS-DA model as assessed by cross validation. In plot B, the blue and red squares indicate the lower and higher metabolite concentrations, respectively. Cx: Acylcarnitines of x chain length; PC: Phosphatidylcholine; SM: Sphingomyelin; lysoPC: Lysophosphatidylcholine. The specific abbreviations of these metabolites are available in Additional file 1: Table S1

In contrast to the similar metabolite profiles observed the CON group, a clear discrimination of metabolite profiles was observed in the scores plot of PLS-DA in the CAR cows before and after the LPS challenge (Fig. 4A). In total, 16 metabolites were classified as important indicators (VIP scores > 1.5) in the first component of the PLS-DA model (Fig. 4B), including 3 acyl-carnitines (C5, C16, and C18), 6 biogenic amines or amino acids (dopamine, ornithine, putrescine, SDMA, spermine, and threonine), 2 sphingomyelins (SM C20:2 and SM OH C24:1) and 3 glycerophospholipids (PC aa C32:1, PC aa C34:1, and PC ae C40:2). However, the exact chemical formula of these sphingomyelins and glycerophospholipids were not available by the current analytical technique. The Q^2^ of the PLS-DA model to discriminate hepatic metabolite profiles was 0.63 (Fig. 4C).

The metabolites identified with the highest VIP scores were further analyzed by linear model or mixed model to assess the effect of carnitine treatment, LPS challenge, and their interaction. All acyl-carnitines of high VIP scores were significantly affected by the carnitine supplementation, and the majority of them were also affected by the LPS challenge, except for acetyl- and propionyl-carnitine (Table 2). Considering biogenic amines and amino acids, supplementary carnitine significantly affected dopamine, while the LPS challenge significantly affected five metabolites in this class (ornithine, putrescine, SDMA, spermine, and threonine) that were classified as important indicators by the PLS-DA model (all except for dopamine) (Table 2).

**Table 2:** Selected hepatic metabolite concentrations of cows treated with or without dietary carnitine

Metabolites, nmol/g	CAR group		SEM	CON group		SEM	*P*-value		
	Before LPS	After LPS		Before LPS	After LPS		LPS	CAR	Interaction
Acyl-carnitines									
C2	39.9	35.3	5.28	10.5	32.6	9.33	0.32	< 0.01	NI^1^
C3	17.7	22.0	2.37	6.05	7.17	1.21	0.61	< 0.01	NI
C4	1.22	1.72	0.14	0.68	1.00	0.14	< 0.01	< 0.01	NI
C5^2^	1.91	2.99	0.26	1.00	1.56	0.33	0.03	< 0.01	NI
C14	0.45	0.59	0.03	0.32	0.56	0.04	< 0.01	< 0.01	NI
C16	0.99	1.58	0.13	0.47	0.14	0.17	< 0.01	< 0.01	NI
C18	1.34	2.03	0.16	0.66	1.25	0.15	< 0.01	< 0.01	< 0.01
Biogenic amines and amino acids									
Dopamine^2^	2.83	1.50	0.41	2.92	4.10	0.71	0.27	0.04	0.08
Ornithine	288.9	212.5	13.0	269.5	193.0	18.9	< 0.01	0.42	NI
Putrescine^2^	3.56	8.73	1.10	5.16	7.81	1.08	< 0.01	0.63	NI
SDMA	0.80	0.35	0.09	0.74	0.52	0.13	< 0.01	0.92	NI
Spermine^2^	10.8	17.1	0.31	12.3	18.3	1.00	< 0.01	0.63	NI
Threonine^2^	494.3	255.5	37.9	435.3	404.5	50.8	< 0.01	0.33	0.05

^1^*NI* Not included in the mixed/linear model due to the non-significant interaction *P*-value (> 0.10)

^2^C5, dopamine, putrescine, spermine, and threonine were fitted by the linear model, while the rest of the parameters were fitted by the mixed model

*CAR* Carnitine-treated, *CON* Control, *Cx* Acylcarnitine of x carbon chain length, *SDMA* Symmetric dimethylarginine

‘Before LPS’ refers to lactation d 100, while ‘after LPS’ refers to lactation d 112

## Discussion

A greater carnitine availability due to dietary carnitine supplementation was associated with altered hepatic metabolite profiles of the cows, including an increased hepatic concentration of short- and long-chain acyl-carnitines and their derivatives. The concentrations of all these acyl-carnitines (C2, C3, C4, C5, C14, C16, and C18) were 3 to sixfold higher in the CAR group than in the CON group, confirming our hypothesis that these intermediary products would accumulate in the liver in relation to enhanced carnitine availability. In the same trial, cows treated with carnitine had significantly higher expression of hepatic very long-chain acyl-CoA dehydrogenase mRNA (*P* < 0.01) [14], suggesting an upregulated utilization of fatty acids with 14–18 carbons in the first step of beta-oxidation. Further, our metabolomics assay revealed the perturbations of hepatic acyl-carnitine, biogenic amine, and amino acid concentrations in association with a LPS challenge in mid-lactation cows. Although previous studies have used metabolomics to study hepatic metabolite profiles of dairy cows [22, 23], the current study was the first to report the alterations of hepatic metabolite profiles associated with supplementary carnitine and LPS injection. Many aspects of the metabolic disturbances that commonly occur in cows during the transition from gestation to lactation are tracked back to an unresolved and often subclinical inflammation, which can involve disturbed mitochondrial function [8, 9]. The metabolic stress of early lactation cows often derives from Gram-negative bacterial mastitis when the blood-milk barrier is disrupted by inflammatory mediators, or from the gastrointestinal bacterial load when the epithelial barrier is disrupted due to subacute ruminal acidosis or “leaky gut” induced by heat stress or other challenges [9]. In addition to these sources of LPS load, the increased circulating concentration of saturated fatty acids, common in early lactation cows, can also activate Toll-like receptor 4 (also known as the LPS receptor), initiating nuclear factor kappa B inflammatory signaling [8]. We chose to use mid-lactation cows in our current study to avoid the common metabolic disturbances of cows directly after calving and during early lactation, so that we can attribute the observed metabolic alterations to the LPS challenge with higher confidence.

Our findings of increased hepatic carnitine concentration were consistent with our previous report where plasma and milk carnitine concentrations of the same cows increased after carnitine supplementation [10]. The uptake of carnitine from the gastrointestinal tract can first increase the carnitine concentration in the hepatic tissues, followed by blood and subsequently milk concentration changes. Supplementary carnitine was shown to enhance fatty acid oxidation in the liver, and decrease BHB and glucose output as well as triglyceride accumulation in the liver of cows [3]. Acyl-carnitines are metabolic byproducts of mitochondrial fatty acid, glucose, and amino acid oxidation [24]; hence they were characterized as biomarkers of mitochondrial functionality in humans [25]. Moreover, circulating concentrations of acyl-carnitines were also linked to insulin resistance in humans [26] and mitochondrial haplotypes of greater oxidative capacity in cows [27]. The liver is discussed to be the main source of circulating acyl-carnitines, and, commonly, studies reporting circulating concentrations interpret their findings as an indication of altered hepatic mitochondrial function [11]. The mitochondrial carnitine shuttle system transports activated long-chain fatty acids from the cytosol inside the mitochondria for subsequent beta-oxidation [1]. The liver can also re-partition the excess of acyl-carnitines to other peripheral tissues, such as to the mammary gland, supporting effective oxidative energy production [28]. In our previous study, cows with improved health status had a higher concentration of carnitine and several acyl-carnitines, such as valeryl-carnitine and hexadecadienyl-carnitine, compared with cows of shorter productive life span [28]. The greater availability of hepatic acyl-carnitines can reduce oxidative stress by avoiding the accumulation of reactive oxygen species. The synthesis of acyl-carnitines prevents CoA trapping, which causes the damage of hepatic cells by the accumulation of fatty acid degradation intermediates, therefore, acyl-carnitine efflux may serve as a detoxification process to protect hepatic mitochondria [11]. As a rate-limiting step, additional carnitine was hypothesized to enhance the transport capacity of long-chain fatty acids into the mitochondria. However, not all transported fatty acids can be fully oxidized in the mitochondria if the transport capacity exceeds oxidation capacity. We found greater hepatic C16 and C18 acyl-carnitine concentrations compared with lower chain lengths such as C14, which can indicate that palmitic acid (16 carbons) and stearic acid (18 carbons) were preferentially transported into the mitochondria by carnitine. This was in line with previous studies showing that the majority of palmitate was esterified in cows treated with supplementary carnitine [5]. The enhanced beta-oxidation of palmitic acid can increase the concentration of acetyl-CoA in mitochondria, stimulating the flux of lactate and alanine through pyruvate carboxylase rather than pyruvate dehydrogenase [3]. The final products of beta-oxidation are acetyl groups that are used in the TCA cycle for energy production. The concentration of acetyl-carnitine (C2) in the CAR group was 4 times higher than its concentration in the CON group, indicating a maximum load of TCA cycle in the mitochondria in the CAR group. Consequently, the concentration of intermediate metabolites related to the rate-limiting steps in the TCA cycle was possibly higher in the CAR group than the CON group too, however, these metabolites were not covered in our metabolomics analysis. Our metabolomics data show that the systemic effect of supplementary carnitine extends to mitochondrial oxidative capacity, even if this could not be reflected by circulating concentrations of conventional biomarkers, such as FFAs, BHB, and TG [19]. In our mid-lactation cows, the concluding results were that the serum concentrations of FFAs, BHB, and TG 24 h after LPS injection were not altered by dietary carnitine supplementation. However, 48–72 h after LPS injection, the concentrations of both serum FFAs and BHB were different between CON and CAR groups [20]. Even if continuous carnitine supplementation does not alter the concentration of these three serum metabolites (FFAs, BHB, and TG) during mid-lactation, their concentrations seem to be affected by the association between lipid metabolism and inflammatory response. Although the stressed condition of hepatic lipid metabolism of cows in early lactation is typically diminished in mid-lactation, the inflammatory response caused by the LPS challenge could still affect the general metabolism in the liver and peripheral tissues, which explained the significant effect of LPS on serum FFAs, BHB, and TG, and hepatic TG [20].

Compared with the significant effect of the carnitine supplementation on acyl-carnitines, its effect on biogenic amines was limited. In fact, only dopamine was affected by the carnitine supplementation, having a decreased concentration after the LPS challenge. Dopamine is primarily synthesized from phenylalanine and tyrosine [29]; however, the concentration of phenylalanine and tyrosine were not affected in our study. Higher dopamine concentrations can reduce prolactin levels, and thereby reduce milk production [30]. A decrease in hepatic dopamine concentration in the carnitine supplemented cows herein is proposed to be a potentially beneficial mechanism to support milk production [19].

The purpose of the intravenously administered LPS was to provoke an inflammatory response, which is a widely used technique to mimic the clinical symptoms caused by an increased LPS load derived from Gram-negative bacteria [31, 32]. The short-term effects of the LPS injection (2.5 to 12 h) on hepatic inflammation and lipid metabolism-related mRNA expressions, such as tumor necrosis factor-alpha and acetyl-CoA carboxylase, were reported previously [14, 33]. On a molecular signaling level, a LPS load above a certain threshold activates inflammatory signaling through a positive feedback loop of nuclear factor kappa B amplification activated via pattern recognition receptors such as Toll-like receptor 4 expressed on Kupffer cells in the liver [8]. In addition, there are also other converging signaling pathways, such as c-Jun NH_2_-terminal kinase and cluster of differentiation 14 mediated signaling, that trigger the release of inflammatory cytokines such as tumor necrosis factor-alpha and interleukin 1 beta from the Kupffer cells as well as the production of acute-phase proteins in the hepatocytes [12]. There are numerous ways in which inflammatory signals can interact with metabolic, translational, and cell cycle regulatory mechanisms [34], which includes potential interactions with liver homeostasis as reviewed by Robinson et al. [35]. Further, we characterized the changes of plasma and milk metabolite profiles 72 h after the LPS injection, and found that the LPS challenge altered plasma and milk short-chain acyl-carnitine concentrations as well as plasma sarcosine, glutamine, and isoleucine concentrations [10]. Our previous studies used serum metabolite profiles to explore the metabolic pathways related to an enhanced productive life span [28] and dietary carnitine supplementation [10] in dairy cows. Both productive life span and carnitine treatment were suggested to be associated with hepatic lipid metabolism and mitochondrial function. At the onset of lactation, the liver plays a central role in the respective homeorhetic adaptations [36], and is also involved in important immune and endocrine functions [37, 38]. The metabolic response to the LPS challenge was different between the carnitine supplemented and the controls cows. In the CON group, acyl-carnitines (mainly C14, C16, C18, and their derivatives) dominated the list of most affected metabolites. Their decreased hepatic concentrations after the LPS challenge likely reflected an enhanced utilization of fatty acids shuttled into the mitochondria for oxidation, and also an increased release of acyl-carnitines from the liver into the circulation, as reflected by increased plasma and milk acyl-carnitine concentrations in these cows reported previously [10]. The latter could reflect passive leak from hepatocytes as well as an active process aiming at the trafficking of acyl-carnitines as either substrates or signal molecules to peripheral tissues.

In contrast, the CAR group showed increased concentrations of C16 and C18 acyl-carnitines after the LPS challenge, indicating well-functioning mitochondria that are in excess of oxidative substrates. Further, the concentrations of biogenic amines (including putrescine and spermine) increased in the liver of carnitine supplemented cows after the LPS challenge, most likely representing an improved anti-oxidant and anti-inflammatory status.

Compared with the effect of the LPS challenge on plasma metabolite profile in our previous study [10], the effects on hepatic metabolite profiles were less prominent herein. The possible explanation is that the LPS challenge likely affected the metabolism of peripheral tissues, such as the mammary gland and skeletal muscle, in addition to the liver. The acute LPS challenge may also affect the interaction between the inflammatory response and oxidative stress response [38], however, reactive oxygen metabolites were not assessed in our analyses. According to our results, the significant effect of the LPS challenge and supplementary carnitine on the majority of acyl-carnitines indicate the potential association between inflammatory response and mitochondrial fatty acid metabolism. In previous studies, the greater extent of lipid mobilization and oxidation for energy production was associated with an increase in the inflammatory response in cows during early lactation [39]. The LPS injection affected the hepatic mRNA expression of genes involved in fatty acid metabolism [14]. In bovine endothelial cells, palmitic acid and stearic acid were shown to promote inflammation by enhancing the production of inflammatory cytokines and reactive oxygen species [40]. The relationship between the inflammatory response triggered by the acute LPS load and the modulation of hepatic metabolic pathways might also be reflected by the observed perturbations in biogenic amine and amino acid metabolism.

Spermine, a polycationic aliphatic amine (polyamine), is synthesized by somatic cells and also by gut microbiota. Polyamines were suggested to increase longevity by decreasing low-grade inflammation in the gut and other organs [41]. The capability of spermidine to modulate autophagy, a mechanism responsible for the regeneration of cellular components and organelles, is discussed to be the major underlying pathway for longevity and other beneficial effects of spermidine [42]. Since polyamines can be also modulated by dietary factors directly or indirectly by modulating microbial populations and their polyamine production, these metabolites are not only proposed as candidates as biomarkers for metabolic balance but also as a target for nutraceutical approaches to improve anti-inflammation and to extend productive life span in dairy cows. As an essential amino acid, threonine could be limiting for optimal protein synthesis in dairy cows [43]. The decrease in hepatic threonine concentrations after the LPS challenge can indicate that interventions supporting protein synthesis might be of interest in an attempt to attenuate inflammatory challenges in cows. This is also supported by the decrease in hepatic ornithine and acetyl-ornithine concentrations, which also point to enhanced proteolysis after LPS challenge, associated with increased urea production and increased metabolism of ornithine to avoid a toxic accumulation of ammonia [44]. The LPS challenge also increased the concentration of conventional blood metabolites, including FFAs, BHB, and triglycerides. Our results were consistent with previous studies in which increased circulating concentrations of FFAs and BHB were observed in cows infused with LPS during mid-lactation [45], which can be explained by the enhanced adipose tissue lipolysis triggered by the inflammatory response [39].

## Conclusions

We showed herein how supplementary carnitine can alter the hepatic metabolome profiles of dairy cows, in particular by increasing the hepatic concentrations of short- and long-chain acyl-carnitines, which are associated with the role of carnitine on hepatic fatty acid metabolism. The inflammatory response triggered by LPS injection altered the hepatic metabolite profiles more prominently in the carnitine supplemented cows, changing acyl-carnitine as well as biogenic amine and amino acid concentrations, including dopamine, ornithine, putrescine, SDMA, spermine, and threonine concentrations. Our findings provide new insight into hepatic metabolic alterations triggered by inflammatory signals, and highlight relationships between metabolic pathways that can be useful for future strategies aiming to attenuate the negative effects of inflammation on hepatic metabolic health.

## Supplementary Information

**Additional file1:** **Table S1.** List of metabolites measured bythe Absolute-IDQ p180 Kit.

## Data Availability

Original data are available at the corresponding author upon reasonable request.

## Abbreviations

BHB: β-Hydroxybutyrate
Cx: Acylcarnitine of x carbon chain length, e.g. C2 Acetyl-carnitine
CAR: Carnitine treatment
CON: Control treatment
FFA: Free fatty acid
LC–MS: Liquid chromatography-mass spectrometry
LPS: Lipopolysaccharide
PLS-DA: Partial least squares-discriminant analysis
Q^2^: Predictive ability of the partial least squares-discriminant analysis model
R^2^: Goodness of fit of the partial least squares-discriminant analysis model
SDMA: Symmetric dimethylarginine
TG: Triglycerides
VIP: Variable importance in projection
