# Supplementary material for: Changes of the liver metabolome following an intravenous lipopolysaccharide injection in Holstein cows supplemented with dietary carnitine
Source: J Anim Sci Biotechnol. 2022 Aug 10;13:94. doi: 10.1186/s40104-022-00741-z (PMC9364515; doi:10.1186/s40104-022-00741-z)
Supplement: Supplementary file 1 — Additional file1: Table S1. List of metabolites measured bythe Absolute-IDQ p180 Kit. [file 40104_2022_741_MOESM1_ESM.docx]

# Supplementary material

**Additional File 1: Table S1.** List of metabolites measured by the Absolute-IDQ p180 Kit

| **Compound class** | | **Metabolites** |
| --- | --- | --- |
| A) | Acyl-carnitines | Carnitine (C0), Acetyl-carnitine (C2), Propionyl-carnitine (C3), Propenoyl-carnitine (C3:1), Hydroxypropionyl-carnitine (C3-OH), Butyryl-carnitine (C4), Butenyl-carnitine (C4:1), Hydroxybutyryl-carnitine (C4-OH (C3-DC)), Valeryl-carnitine (C5), Tiglyl-carnitine (C5:1), Glutaconyl-carnitine (C5:1-DC), Glutaryl-carnitine (Hydroxyhexanoyl-carnitine) (C5-DC (C6-OH)), Methylglutaryl-carnitine (C5-M-DC), Hydroxyvaleryl-carnitine (Methylmalonyl-carnitine) (C5-OH (C3-DC-M)), Hexanoyl-carnitine (Fumaryl-carnitine) (C6 (C4:1-DC)), Hexenoyl-carnitine (C6:1), Pimelyl-carnitine (C7-DC), Octanoyl-carnitine (C8), Nonayl-carnitine (C9), Decanoyl-carnitine (C10), Decenoyl-carnitine (C10:1), Decadienyl-carnitine (C10:2), Dodecanoyl-carnitine (C12), Dodecenoyl-carnitine (C12:1), Dodecanedioyl-carnitine (C12-DC), Tetradecanoyl-carnitine (C14), Tetradecenoyl-carnitine (C14:1), Hydroxytetradecenoyl-carnitine (C14:1-OH), Tetradecadienyl-carnitine (C14:2), Hydroxytetradecadienyl-carnitine (C14:2-OH), Hexadecanoyl-carnitine (C16), Hexadecenoyl-carnitine (C16:1), Hydroxyhexadecenoyl-carnitine (C16:1-OH), Hexadecadienyl-carnitine (C16:2), Hydroxyhexadecadienyl-carnitine (C16:2-OH), Hydroxyhexadecanoyl-carnitine (C16-OH), Octadecanoyl-carnitine (C18), Octadecenoyl-carnitine (C18:1), Hydroxyoctadecenoyl-carnitine (C18:1-OH), Octadecadienyl-carnitine (C18:2) |
| B) | Amino acids | Alanine (Ala), Arginine (Arg), Asparagine (Asn), Aspartate (Asp), Citrulline (Cit), Glutamine (Gln), Glutamate (Glu), Glycine (Gly), Histidine (His), Isoleucine (Ile), Leucine (Leu), Lysine (Lys), Methionine (Met), Ornithine (Orn), Phenylalanine (Phe), Proline (Pro), Serine (Ser), Threonine (Thr), Tryptophan (Trp), Tyrosine (Tyr), Valine (Val) |
| C) | Biogenic amines | Acetyl-ornithine (Ac-Orn), Asymmetric dimethyl-arginine (ADMA), Symmetric dimethyl-arginine (SDMA), alpha-Aminoadipic acid (alpha-AAA), Carnosine (Carnosine), Creatinine (Creatinine), Histamine (Histamine), Kynurenine (Kynurenine), Methionine sulfoxide (Met-SO), Nitro-tyrosine (Nitro-Tyr), cis-4-Hydroxyproline (cis-OH-Pro), trans-4-Hydroxyproline (trans-OH-Pro), Phenylethylamine (PEA), Putrescine (Putrescine), Sarcosine (Sarcosine), Serotonin (Serotonin), Spermidine (Spermidine), Spermine (Spermine), Taurine (Taurine), Dopamine (Dopamine), DOPA (DOPA) |
| D) | Glycerophospholipids |  |
|  | Lyso-PC | lysoPC a C14:0, lysoPC a C16:0, lysoPC a C16:1, lysoPC a C17:0, lysoPC a C18:0, lysoPC a C18:1, lysoPC a C18:2, lysoPC a C20:3, lysoPC a C20:4, lysoPC a C24:0, lysoPC a C26:0, lysoPC a C26:1, lysoPC a C28:0, lysoPC a C28:1 |
|  | Diacyl-PC | PC aa C24:0, PC aa C26:0, PC aa C28:1, PC aa C30:0, PC aa C30:2, PC aa C32:0, PC aa C32:1, PC aa C32:2, PC aa C32:3, PC aa C34:1, PC aa C34:2, PC aa C34:3, PC aa C34:4, PC aa C36:0, PC aa C36:1, PC aa C36:2, PC aa C36:3, PC aa C36:4, PC aa C36:5, PC aa C36:6, PC aa C38:0, PC aa C38:1, PC aa C38:3, PC aa C38:4, PC aa C38:5, PC aa C38:6, PC aa C40:1, PC aa C40:2, PC aa C40:3, PC aa C40:4, PC aa C40:5, PC aa C40:6, PC aa C42:0, PC aa C42:1, PC aa C42:2, PC aa C42:4, PC aa C42:5, PC aa C42:6 |
|  | Acyl-alkyl-PC | PC ae C30:0, PC ae C30:1, PC ae C30:2, PC ae C32:1, PC ae C32:2, PC ae C34:0, PC ae C34:1, PC ae C34:2, PC ae C34:3, PC ae C36:0, PC ae C36:1, PC ae C36:2, PC ae C36:3, PC ae C36:4, PC ae C36:5, PC ae C38:0, PC ae C38:1, PC ae C38:2, PC ae C38:3, PC ae C38:4, PC ae C38:5, PC ae C38:6, PC ae C40:1, PC ae C40:2, PC ae C40:3, PC ae C40:4, PC ae C40:5, PC ae C40:6, PC ae C42:0, PC ae C42:1, PC ae C42:2, PC ae C42:3, PC ae C42:4, PC ae C42:5, PC ae C44:3, PC ae C44:4, PC ae C44:5, PC ae C44:6 |
| (E) | Sphingolipids | SM (OH) C14:1, SM (OH) C16:1, SM (OH) C22:1, SM (OH) C22:2, SM (OH) C24:1, SM C16:0, SM C16:1, SM C18:0, SM C18:1, SM C20:2, SM C22:3, SM C24:0, SM C24:1, SM C26:0, SM C26:1 |

*PC*: phosphatidylcholines; *SM*: sphingomyelins.
